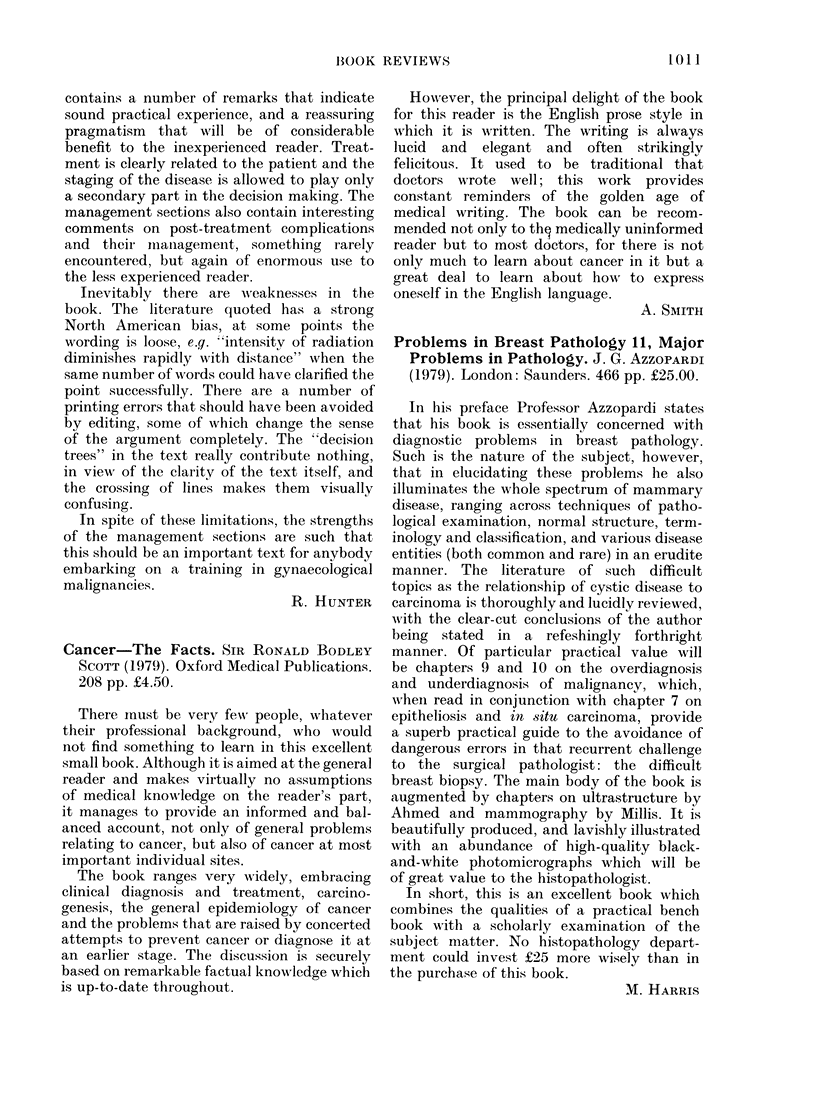# Cancer—The Facts

**Published:** 1980-06

**Authors:** A. Smith


**Cancer—The Facts.** SIR RONALD BODLEY SCOTT (1979). Oxford Medical Publications. 208 pp. £4.50.

There must be very few people, whatever their professional background, who would not find something to learn in this excellent small book. Although it is aimed at the general reader and makes virtually no assumptions of medical knowledge on the reader's part, it manages to provide an informed and balanced account, not only of general problems relating to cancer, but also of cancer at most important individual sites.

The book ranges very widely, embracing clinical diagnosis and treatment, carcinogenesis, the general epidemiology of cancer and the problems that are raised by concerted attempts to prevent cancer or diagnose it at an earlier stage. The discussion is securely based on remarkable factual knowledge which is up-to-date throughout.

However, the principal delight of the book for this reader is the English prose style in which it is written. The writing is always lucid and elegant and often strikingly felicitous. It used to be traditional that doctors wrote well; this work provides constant reminders of the golden age of medical writing. The book can be recommended not only to the medically uninformed reader but to most doctors, for there is not only much to learn about cancer in it but a great deal to learn about how to express oneself in the English language.

A. SMITH